# Supplementary material for: Idebenone Mitigates Traumatic-Brain-Injury-Triggered Gene Expression Changes to Ephrin-A and Dopamine Signaling Pathways While Increasing Microglial Genes
Source: Cells. 2025 Jun 1;14(11):824. doi: 10.3390/cells14110824 (PMC12154110; doi:10.3390/cells14110824)
Supplement: Supplementary file 1 [file cells-14-00824-s001.zip › Supplemental Table S3.pdf]

Supplemental Table S3. Complete list of genes differentially expressed between the TBI+vehicle and TBI +idebenone groups with p-values between 0.05 and 0.1. The genes shown in bold encode proteins reported to interact with the idebenone-binding protein SHC1, or with SHC1-interacting receptors. The underlined genes are preferentially expressed by microglia within the healthy mouse brain [19].

Name	Description	Fold Change	p-Value
Adora2a	adenosine A2a receptor	4.23	0.057
**Ntrk1**	neurotrophic tyrosine kinase, receptor, type 1	2.12	0.062
Adcy5	adenylate cyclase 5	1.82	0.057
Pde1b	phosphodiesterase 1B, Ca2+-calmodulin dependent	1.66	0.068
Gabra4	gamma-aminobutyric acid (GABA) A receptor, subunit alpha 4	1.29	0.067
<u>Irf8</u>	interferon regulatory factor 8	1.28	0.076
Ccnd1	cyclin D1	1.28	0.095
Nol3	nucleolar protein 3 (apoptosis repressor with CARD domain)	1.27	0.075
<u>Nfkbia</u>	nuclear factor of kappa light polypeptide gene enhancer in B cells inhibitor, alpha	1.23	0.096
<u>C1qc</u>	complement component 1, q subcomponent, C chain	1.23	0.050
<u>P2rx4</u>	purinergic receptor P2X, ligand-gated ion channel 4	1.22	0.079
Kcna1	potassium voltage-gated channel, shaker-related subfamily, member 1	1.20	0.086
S100b	S100 protein, beta polypeptide, neural	1.19	0.081
Eng	endoglin	1.18	0.088
Ppp3ca	protein phosphatase 3, catalytic subunit, alpha isoform	1.18	0.059
<u>Cx3cr1</u>	chemokine (C-X3-C motif) receptor 1	1.15	0.070
Gnb5	guanine nucleotide binding protein (G protein), beta 5	1.14	0.088
**Igf1r**	insulin-like growth factor I receptor	1.13	0.061
Cntnap2	contactin associated protein-like 2	1.13	0.093
Nfe2l2	nuclear factor, erythroid derived 2, like 2	1.13	0.085
Mapt	microtubule-associated protein tau	1.12	0.072
Glrb	glycine receptor, beta subunit	1.12	0.055
**Src**	Rous sarcoma oncogene	1.12	0.099
Chd4	chromodomain helicase DNA binding protein 4	1.11	0.090
**Rhoa**	ras homolog gene family, member A	1.11	0.060
**App**	amyloid beta (A4) precursor protein	1.07	0.058
Dld	dihydrolipoamide dehydrogenase	-1.06	0.098
Nell2	NEL-like 2	-1.07	0.072
Il6	interleukin 6	-1.25	0.094
Casp6	caspase 6	-1.26	0.099
**Ngf**	nerve growth factor	-1.50	0.072
Epha3	Eph receptor A3	-1.71	0.078
