# Supplementary material for: Idebenone Mitigates Traumatic-Brain-Injury-Triggered Gene Expression Changes to Ephrin-A and Dopamine Signaling Pathways While Increasing Microglial Genes
Source: Cells. 2025 Jun 1;14(11):824. doi: 10.3390/cells14110824 (PMC12154110; doi:10.3390/cells14110824)
Supplement: Supplementary file 1 [file cells-14-00824-s001.zip › Supplemental Table S4.pdf]

Supplemental Table S4. Results of "ENCODE and ChEA Consensus TFs from ChIP-X" Enrichr query for idebenone-affected genes following expansion of the list to include the *Drd2*-correlated genes in Figure 10a-c. The adjusted p-value (p-adj) was calculated using the Benjamini-Hochberg method for correction for multiple hypotheses testing.

term	p-value	p-adj value	overlap_genes
**SUZ12** ChEA	3.752209e-12	2.101237e-10	Adcy5, Adora2a, Bcl2, Calb1, Camk4, Cxcl12, Ccxr4, Drd2, Efna5, Epha3, Epha5, Epha6, Epha7, Gabra4, Gad2, Htr1a, Mal, Mmp9, Negr1, Npy, Ntf3 Slc32a1
**ESR1** ChEA	0.000466	0.012189	Bcl2, Cxcl12, Efna1, Itpr1
**REST** ChEA	0.000653	0.012189	Adcy5, Calb1, Drd1, Drd2, Fgf14, Gad2, Htr1a, Slc28a3, Slc32a1, Tenm2
**REST** ENCODE	0.001988	0.027833	Drd2, Calb1, Chat, Grm2, Htr1a
**EZH2** ChEA	0.018165	0.203448	Adcy5, Camk4, Gad2
**AR** ChEA	0.042033	0.392305	Ang, Efna1
