# Supplementary figures and images for: Idebenone Mitigates Traumatic-Brain-Injury-Triggered Gene Expression Changes to Ephrin-A and Dopamine Signaling Pathways While Increasing Microglial Genes
Source: Cells. 2025 Jun 1;14(11):824. doi: 10.3390/cells14110824 (PMC12154110; doi:10.3390/cells14110824)

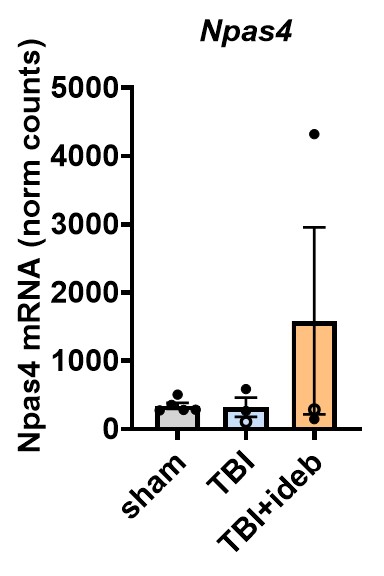

Supplement: Supplementary file 1 [file cells-14-00824-s001.zip › Supplemental Figure S1.jpg]
